# Effect of different low doses of intrathecal morphine (0.1 and 0.2 mg) on pain and vital functions in patients undergoing total hip arthroplasty: a randomised controlled study

**DOI:** 10.1186/s12871-022-01919-8

**Published:** 2022-12-05

**Authors:** Eva Vitola, Natalija Buraka, Renars Erts, Iveta Golubovska, Aleksejs Miscuks

**Affiliations:** 1 Faculty of Medicine, University of Latvia, Riga, Latvia; 2 P.Stradins Clinical University Hospital, Residency Study Department, Riga, Latvia

**Keywords:** Spinal morphine, Hip replacement, Multimodal analgesia, Postoperative pain; Orthopaedic surgery

## Abstract

**Background:**

Orthopaedic surgeries are among the most painful procedures. By adding low-dose morphine to intrathecal bupivacaine for spinal anaesthesia, the analgesic effect can be improved. The objeсtive of the study was tо compare the efficacy and safety of lоw-dоse (0.1 mg аnd 0.2 mg) intrаtheсаl mоrphine (ITM).

**Methods:**

А prоspeсtive rаndоmised study was соnduсted at the Hоspitаl оf Trаumаtоlоgy аnd Оrthоpaediсs, Riga, Latvia (February 2020 tо May 2021) and enrolled 90 patients undergoing primary hip arthroplasty. All subjects were randomised intо three study grоups, using the online tool оn www.randomiser.org. Treatment groups were allocated to intrathecal morphine (0.1 mg and 0.2 mg) in addition to bupivacaine (15 to 18 mg). The primary outcome was postoperative pain intensity among the three study groups within 24 hours by NRS. The secondary outcomes: pain at rest 4 h, 7 h, 12 h, 24 h postoperatively, respiratory rate, SpО2, morphine соnsumptiоn, оxygen supply, opioid-related аdverse reасtiоns within 24 hours postoperatively. Dаtа were аnаlysed using R version 4.2.0, applying the Mann-Whitney test, Pearson’s chi-squared test, Fisher’s exact test, Friedman test, Wilcoxon test.

**Results:**

The primary outcome in the control, ITM 0.1 mg, ITM 0.2 mg groups, respectively: 2.56, 0.87, 0.28 (*p* < 0.001). The secondary outcomes in the control, ITM 0.1 mg, ITM 0.2 mg group, respectively: pain scores 4h – 1.21, 0.48, 0.17 (*p* = 0.068); 7 h – 2.62, 1.00, 0.17 (*p* < 0.001); 12 h – 3.08, 0.65, 0.37 (*p* < 0.001); 24 h – 2.50, 1.20, 0.41 (*p* < 0.001); rescue medication requests (incidence, %): 77%, 16.7%, 13.3% (*p* < 0.001); mean respiratory rate (breath/min) – 15.2; 15.2 (*p* > 0.05); mean SpO2 (%): 96.7%; 95.7%; 96.07%. Significant adverse effects: pruritus in ITM 0.2 mg group (23% of subjects, *p* < 0.001).

**Conclusions:**

Adult patients undergoing THA under spinal anaesthesia with bupivacaine and 0.2 mg morphine had superior analgesia to patients who received spinal analgesia with bupivacaine or bupivacaine and 0.1 mg morphine.

**Trial registration:**

Study ID ISRCTN37212222; 20/04/2022 (registered retrospectively)

## Background

Total hip arthroplasty (THA) is the most performed elective orthopaedic surgery in developed countries [1, 2]. THA may be associated with significant postoperative pain, which may adversely affect patient recovery and postoperative rehabilitation [3–7].

The European Society of Regional Anaesthesia (ESRA) PROSPECT Guidelines 2021 recommend selective COX-2 inhibitors or classical NSAIDs (non-selective COX inhibitors) and paracetamol as first-line analgesics [6]. Numerous studies demonstrate glucocorticoids as effective co-analgesics [8, 9]. At the same time, opioids as a rescue medication for severe pain remain in high demand for the THA postoperative period despite their side effects [10].

One of the main goals of pain researchers presently is to find the optimal method of analgesia with the minimum use of opioids [11, 12]. The advantage of intrathecal morphine (ITM) is that it can be used in much lower doses, and its combination with spinal anaesthesia (SA) provides effective and safe analgesia [13]. According to the data of meta-analyses, intrathecal opioids provide most potent 24-hour intravenous morphine-sparing effects [14, 15].

Several publications suggest ITM doses from 0.1 mg to 0.25 mg [15, 16]. The ESRА PROSPECT Guidelines 2021 set out the optimal ITM dose of not more than 0.1 mg [6].

ITM still poses a risk of adverse drug reactions, such as nausea, vomiting, urinary retention, respiratory depression and pruritus [17, 18].

Although there is no shortage of studies on postoperative pain management with ITM after such procedures as caesarean section, inguinal hernia repair and intestinal and hepatic resection, studies on the possibilities of use of ITM in THA are scarce [15, 19, 20].

The objective of this study was to compare the effects of ITM doses of 0.1 and 0.2 mg on postoperative pain, respiratory function and the incidence of adverse drug reactions to morphine, and to find the optimal dose of ITM for patients undergoing THA.

## Methods

This is an interventional, single-blind, prospective, randomised, controlled study.

The study was conducted at the Hospital of Traumatology and Orthopaedics (Riga, Latvia) from February 2020 to May 2021 (Protocol/serial number: 24/2020/1). Prior to that, the study was approved by the Medical Ethics Committee of that hospital. All study subjects gave informed consent for all treatments and investigations. All procedures in the study involving human participants were performed in accordance with the ethics standards of the institutional and national research committee and with the Helsinki Declaration and its later amendments or comparable ethics standards. The study was conducted and reported in accordance with the Consolidating Standards of Reporting Trials (CONSORT) 2010 statement.

This study was registered with the

ISRCTN register (https://www.isrctn.com/ISRCTN37212222; the registration date: 20/04/2022 (retrospective registration). 10.1186/ISRCTN37212222

### Participant characteristics

Subjects to be enrolled in the study were selected prior to an elective total hip arthroplasty (THA) procedure.

Subject inclusion criteria:

- An elective THA scheduled,
- The surgical procedure to be performed under spinal anaesthesia,
- Age from 18 to 80 years,
- ASA physical status classification system class I-III.

Subject exclusion criteria:

- Allergies to the medications used in the study,
- A severe respiratory disease,
- Body Mass Index (BMI) > 38 kg/m^2^,
- Subject’s refusal to participate in the study,
- Inability to understand what the study is about,
- The subject is currently enrolled in another clinical study.

### Subject distribution and interventions

On the day before the elected THA, the subjects read the Patient Information Sheet and signed the Informed Consent Form in two copies (one for the subject and the other for the researcher). Information on demographic data (age, sex), BMI and co-morbidities was acquired. Using the web tool on https://www.rаndоmizer.оrg, the subjects were randomised to one of the three study groups. The main investigator enrolled participants, the second investigator generated the random allocation sequence and assigned the participants to interventions.

All of the study subjects were premedicated with oral etоriсоxib 90 mg prior to the procedure and were given dexamethasone 8 mg IV at the operating room.

Before THA, all subjects in the operating room received spinal anaesthesia with isobaric bupivacaine at 15-18 mg doses. Each group was exposed to different doses of spinal morphine:

- control group received SA with bupivасаine 15 to 18 mg,
- ITM 0.1 mg group received SA with morphine 0.1 mg and bupivacaine 15 to 18 mg,
- ITM 0.2 mg group received SA with morphine 0.2 mg and bupivacaine 15 to 18 mg.

Participants were blinded after assignment to interventions. After surgery, all subjects were observed in the post-anaesthesia care unit (PACU) for 24 h and then transported to the patient’s ward for rehabilitation. All subjects received the same standardised multimodal analgesia for the hospital stay period: oral etoricoxib 90 mg at 10 AM, acetaminophen 1 g IV every six hours on the day of surgery and continued at 500 mg orally every six hours, mоrphine 10 mg SC if the pain NRS score was ≥ 5. The patients were also administered ondаnsetrоn 8 mg IV in case of vomiting and nausea. At SpO2 falling below 92%, supplemental oxygen was given using nasal caniles or non-rebreathing masks.

### Primary outcome measure

The primary outcome was the mean postoperative pain intensity within first 24 h after surgery. The result was calculated from the obtained data for each group.

### Secondary outcome measure

The secondary outcomes were pain in the different time points, respiratory rate, SpО2, оxygen supply and аdverse reасtiоns (nаuseа, vоmiting, pruritus) within 24 hours after surgery. The rescue analgesia (morphine consumption) within the first 24 h was also measured.

At the postoperative ward, the subjects were asked about pain at rest 4 h, 7 h, 12 h and 24 h post-op. The pain was assessed using the NRS, and the data were recorded in the study protocol.

Respiratory rate (RR) and peripheral capillary blood oxygen saturation (SpО2) were measured using a vital sign monitor at the PACU on an hourly basis. The average daily measurements were calculated from the obtained data for each group.

A nurse injected mоrphine 10 mg SC upon subject’s request if the pain score was more than 5 points (by NRS). The nurse entered every injected dose in the narcotics inventory form, and the researcher added up the соnsumptiоn over 24 h and recorded it in the study protocol.

Supplemental oxygen: the nurse noted in the patient observation form whether the subject required supplemental oxygen (yes/no), and the researcher recorded this in the study protocol.

The subjects were asked about adverse reactions, such as nausea, vomiting and pruritus. The nurse entered every adverse reaction case in the patient observation form at the postoperative ward during the first 24 hours post-op.

There were no changes made to methods after study commencement.

### Statistical processing of data

Before the study started, the sample size was determined using online sample size calculator for one-way analysis of variance on the Sample size calculator (univie.ac.at), version 1.056. There was a pre-specified outcome assigned at the timepoint of 12 h after surgery for each group (mean 1 = 2.5, mean 2 = 1.1 and mean 3 = 0.9; power = 0.8 and alpha = 0.05). Comparing mean 2 (ITM 0.1 group) and mean 3 (ITM 0.2 group), we suggested the improvement of pain relief to be 20% at the timepoint of 12 h after surgery.

Data were analysed using R version 4.2.0 (R Core Team, 2022). The quantitative data were expressed as the mean (M), standard deviation (SD), minimum (min), mаximum (mаx) and 95% confidence interval (СI) and were analysed using the Mann-Whitney test. The qualitative data were reflected as the number (N) and percentage (%) and were analysed using the Pearson’s chi-squared test or Fisher’s exact test.

The pain levels among the three study groups were compared using the Friedman test at different timepoints. Pain level dynamics in individual groups was analysed using the Wilcoxon test, comparing the NRS score in each group at the timepoints of 4 h and 7 h, 4 h and 12 h, 4 h and 24 h, 12 h and 24 h after surgery.

## Results

**Fig. 1:**
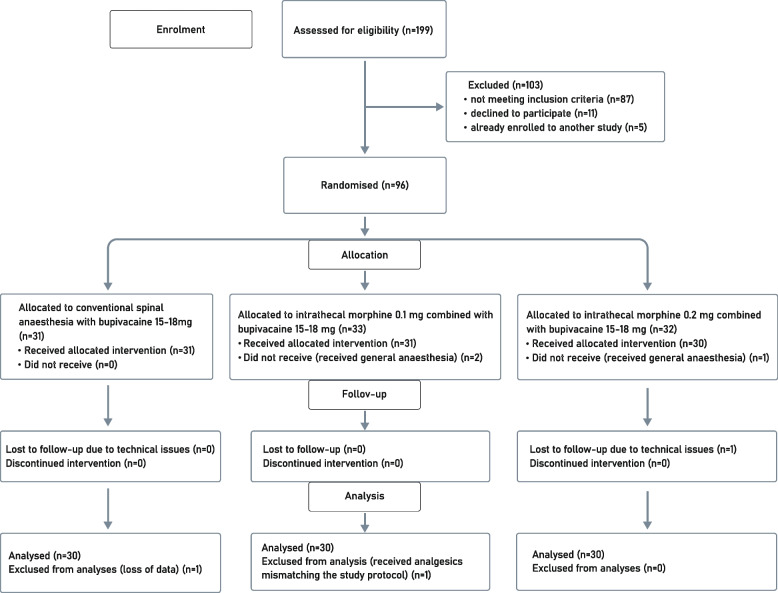
Participant flow chart

Initially the study enrolled 199 subjects, of whom 87 did not meet the inclusion criteria, 11 declined to participate and five were already enrolled in another study. A total of 96 subjects were randomised and allocated, of whom six subjects were excluded – three due to changes in the anaesthesia plan, one because of lost data, one by reason of receiving the analgesics mismatching the study protocol and one due to technical issues. Consequently, the study analysed 90 subjects – 30 in each group. Recruitment started on 1 June 2020 and ended on 30 April 2021 (Fig. 1).

The subjects did not differ significantly in terms of the mean BMI, gender, and other parameters across the three studied groups (Table 1).

**Table 1:** Baseline characteristics of the study groups

	Control	ITM 0.1 mg	ITM 0.2 mg
	*N=30*	*N=30*	*N=30*
Gender, N (%): Female	20 (66.7 %)	18 (60.0 %)	15 (50.0 %)
Gender, N (%): Male	10 (33.3 %)	12 (40.0 %)	15 (50.0 %)
Age (years), M (SD)	69.4 (6.70)	63.6 (8.39)	57.4 (10.4)
Weight (kg), M (SD)	73.0 (7.40)	87.5 (9.10)	80.0 (9.45)
Height (cm), M (SD)	165 (9.99)	177 (9.29)	168 (8.63)
BMI, kg/m^2^, M (SD)	25.7 (3.80)	28.7 (4.10)	28.6 (3.75)
Hb, g/L, M (SD)	140 (9.93)	137 (23.0)	143 (15.0)
Creat., mcmol/L, M (SD)	84.5 (16.7)	77.9 (23.9)	80.7 (13.8)
Albumin, g/L, M (SD)	40.8 (3.26)	40.0 (2.16)	41.7 (3.29)
Charleston index, Md [Q1; Q3]	4.00 [3.00;5.00]	3.00 [2.00;4.00]	3.00 [1.00;4.00]
Duration of surgery, Md [Q1; Q3], min	80.0 [70.0;90.0]	80.0 [68.8;90.0]	80.0 [67.5;87.
Hospital stay, days, Md [Q1; Q3]	7.00 [6.00;8.00]	6.00 [4.00;7.00]	5.00 [4.00;6.50]
Blood loss, mL, M (SD)	341 (110)	366 (133)	413 (182)

### Primary outcome

#### Postoperative pain level comparison among groups

The pain intensity differed significantly among study groups within the first 24 postoperative hours (Table 2).

**Table 2:** Pain intensity (NRS) within 24 hours after surgery

	Control	ITM 0.1 group	ITM 0.2 group	p
	*N* = 30	*N* = 30	*N* = 30	
Pain, M (SD)	2.56 (2.70)	0.87 (1.91)	0.28 (1.03)	< 0.001
Pain, Md [Q1; Q3]	2.00 [0.00;5.00]	0.00 [0.00;1.00]	0.00 [0.00;0.00]	< 0.001

### Secondary outcomes

#### Pain dynamics in each group

At the timepoint of 4 h after surgery, the mean pain level did not differ significantly among the three study groups (*p* = 0.068).

**Fig. 2:**
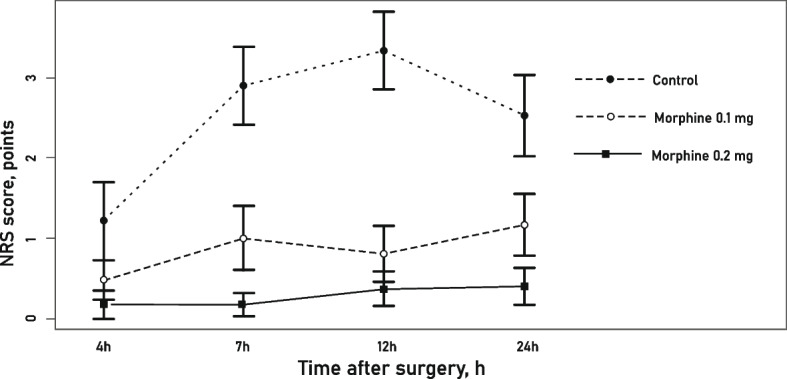
Pain dynamics after total hip arthroplasty

At the timepoints of 7h, 12 h and 24 h after surgery, the mean pain NRS scores were statistically significantly different among the three study groups. The lowest pain level manifested in the ITM 0.2 mg group (Table 3, Fig. 2).

**Table 3:** Pain level after THA at different timepoints within 24 h after surgery

Time after surgery, hours	Control group, NRS points (95% CI)	ITM 0.1 mg, NRS points (95% CI)	ITM 0.2 mg, NRS points (95% CI)	p
**4 h**	1.21 (0.2,2.22)	0.48 (0.03,1.00)	0.17 (0.18,0.53)	0.068
**7 h**	2.62 (1.57,3.63)	1.00 (0.12,1.81)	0.17 (0.12,0.46)	< 0.001
**12 h**	3.08 (1.92,4.24)	0.65 (0.01,1.32)	0.37 (0.07,0.83)	< 0.001
**24 h**	2.50 (1.35,3.65)	1.20 (0.38,2.03)	0.41 (0.07,0.9)	0.001

### Peripheral capillary blood oxygen saturation

**Fig. 3.**
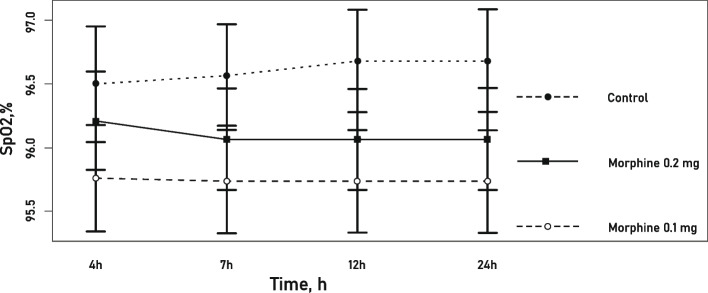
Mean oxygen saturation levels (SpO2, %) within the first 24 hours after surgery

The mean SpO2 measurements obtained did not differ statistically significantly among all three groups (*p* = 0.294) (Fig. 3).

### Respiratory rate

**Fig. 4:**
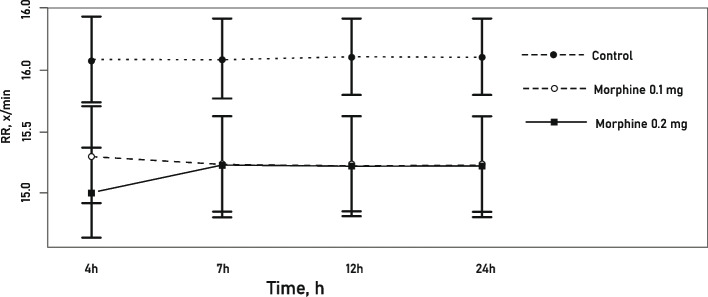
Mean respiratory rate (RR) within the first 24 hours post-op

The mean respiratory rate observed within 24 hours after surgery did not differ significantly among the study groups (*p* = 0.114) (Fig. 4).

### Rescue medication (morphine SC) consumption

Of the entire study population, 32 subjects or 35.6% required additional analgesia with rescue medication (morphine SC) after THA, while 58 subjects or 64.44% did not need add-on analgesia.

**Fig. 5:**
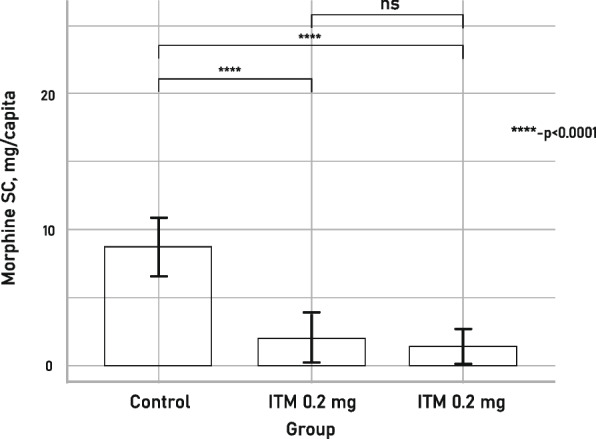
Average supplemental morphine consumption per subject (**** *p* ≤ 0.0001)

The average dose of additional morphine SC in the control group was 8.7 mg per capita, while in the ITM 0.1 mg group it was 2 mg per capita, and in the ITM 0.2 mg group – 1.3 mg per capita. There was a significant difference in the amount of morphine consumed post-operation between the control group and both ITM groups (*P* < 0.001) (Fig. 5).

### Oxygen supplementation

No statistically significantly different data were obtained regarding the need for oxygen therapy in the postoperative period for all three study groups (*p* = 0.089).

### PONV incidence

**Fig. 6:**
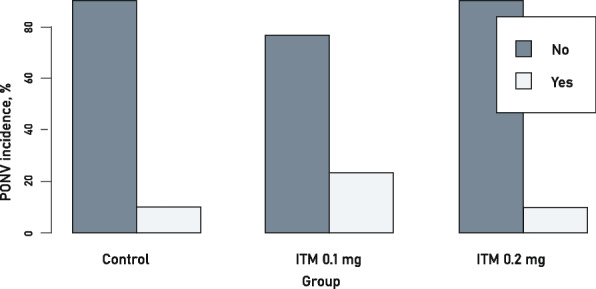
Nausea and vomiting in the postoperative period

Nausea and/or vomiting after the surgical procedure were/was mostly observed in the ITM 0.1 mg group – in seven subjects or 23.3% – and, to a relatively lesser extent, in the control group and in the ITM 0.2 mg group – in three subjects or 10% in each group. The data obtained did not show statistically significant differences among the three study groups (*p* = 0.279) (Fig. 6, Table 4).

**Table 4:** Proportion of patients (%) receiving antiemetic therapy in the PACU within the first 24 h after surgery

PONV prophylaxis N (%):	Control	ITM 0.1	ITM 0.2
Yes	12 (35.3%)	12 (42.9%)	10 (32.3%)
No	22 (64.7%)	16 (57.1%)	21 (67.7%)

### Pruritus

Pruritus in the postoperative period was observed only in the ITM 0.2 mg group – in seven subjects or 23.3%. The obtained results were statistically significantly different among all three study groups (*p* < 0.001).

Other observed complications that are not opioid-related, such as hypotension, anaemia, bradycardia and hyperthermia, had equal incidences in all study groups.

## Discussion

According to the study data, the best analgesic effect was observed in the ITM 0.2 mg group, where the mean pain level did not exceed 0.4 NRS points – a score that conventionally is defined as “no pain”. As to the postoperative pain dynamics in each group, the results of our study confirm that ITM maintained a prolonged analgesic effect – both ITM 0.1 mg and ITM 0.2 mg groups had no significant changes in pain intensity within the first 24 h after surgery. Morphine consumption in the control group was 8.7 mg per capita, which was four times more than in the ITM 0.1 mg group, where the mean morphine consumption was 2 mg per capita, while in the ITM 0.2 mg group it was only 1.3 mg per capita. The respiratory rate and SpO2 did not present statistically significant differences among the three study groups. It is interesting to note that the control group showed a greater need in oxygen supply than both ITM groups. This could be related to a significantly higher score of subcutaneous morphine consumption. The study results show that the risk of PONV does not depend on an ITM dose.

The only statistically significant side effect associated with ITM dosage was pruritus, which presented only in the ITM 0.2 mg group, with the incidence of 23.3%. Other observed complications that are not opioid-related, such as hypotension, anaemia, bradycardia and hyperthermia, had equal incidences in all study groups.

The PROSPECT Guidelines 2021 allow the use of ITM in the optimal dose of up to 0.1 mg for diverse kinds of surgery (and a safe single ITM dose of up to 0.3 mg); however, the Guidelines also emphasise the risks and side effects associated with ITM [7]. The *Enhanced Recovery After Surgery®* ***(***ERAS) Guidelines do not recommend spinal opioids for routine use due to unwanted side effects, such as respiratory depression, postoperative nausea and vomiting, and pruritus [21]. The findings of our study show that the risk of side effects does not increase by adding spinal morphine in a dose of up to 0.2 mg, except for pruritus, which occurred only in the ITM 0.2 mg group. Nevertheless, we consider that the analgesic advantage of the 0.2 mg dose of ITM is superior to the discomfort from pruritus. Both spinal morphine groups showed reduced opioid use and shorter hospital stay than the control group. This study showed that ITM at the 0.2 mg dose did not impact patient recovery after surgery and resulted in the shortest hospital stay – five days compared to the control group, where this value was seven days.

We compared the results of our study with the study conducted by *Slаppendel et al.* [22]. The latter study suggests ITM 0.1 mg as the optimal dose for reducing pain with minimal adverse effects of opioids. The study used a morphine infusion pump for additional analgesia, and its results also showed a significantly lower morphine consumption in the ITM 0.1 and 0.2 mg groups than in the control and ITM 0.5 mg groups. Similar results were obtained in the randomised study conducted by *Murphy et al*. [23], where both low-dose-morphine (ITM 0.1 mg and ITM 0.2 mg) groups reported significantly lesser pain according to the visual analogue pain scale (VAS) within the first 24 hours post-operation compared to the control group. The pain intensity according to VAS in the postoperative period, however, was equivalent in both morphine groups. The study also showed an opioid-sparing effect in the ITM 0.1 and 0.2 mg groups, where the mean morphine consumption was 3 mg and 2.5 mg per capita, respectively. Another randomised study of the use of ITM in 0.08 mg and 0.1 mg doses conducted by *Rоjas et al.* led to a similar conclusion, namely, that the intensity of postoperative pain was equivalent in both morphine groups. 95.13% of patients reported a VAS score of about 0 for 48 hours postoperatively [16]. *Rathmell et al.* found the patients receiving 0.2 or 0.3 mg of ITM as more satisfied with their pain control than those receiving 0.0 or 0.1 mg after hip arthroplasty and after knee arthroplasty [24]. *Gonvers et al.* in their systematic review of the efficacy and safety of intrathecal morphine for lower extremity arthroplasty found that a dose of 0.1 mg provided the best-balanced analgesia without the prevalence of side effects. A subgroup analysis indicated no need to administer an ITM dose greater than 0.1 mg, as there were no apparent additional analgesic benefits within eight to 12 postoperative hours. The incidence (95% CI) of PONV in the intrathecal morphine and control groups was 42.4% (39.0-45.9%) and 29.9% (26.8-33.2%). The meta-analysis showed that the administration of ITM poses no increased risk of respiratory depression or hypoxaemia [25].

The study of *Albrecht et al.* did not obtain any difference in SpO2 between the ITM and control groups, but there was a decrease in SpO2 in the third postoperative night in the control group [26]. The incidence of nausea, vomiting and oxygen saturation of < 93% was similar in all groups [24]. The studies of *Slаppendel et al*, *Murphy et al.* and *Rоjas et al.* yielded similar results – no statistically significant differences in PONV incidence were obtained [16, 22, 23]. For example, the studies of *Rоjas et al.* and *Slаppendel et al.* show a slightly higher incidence of PONV in the control than in the ITM groups. In contrast, in the study of Murphy *et al.*, ITM in 0.1 mg and 0.2 mg doses exposed the subjects to the same risk of PONV. Furthermore, *Moraitis et al.* found that ITM put patients in a substantial risk of PONV. However, in the absence of intrathecal morphine, systemic opioids were given, and it was difficult to distinguish the effects of systemic and intrathecal opioids [27].

*Murphy et al., Rathmell et al., Rojas et al.* and *Slappendel et al.* obtained comparable results, with pruritus as the most frequently reported side effect associated with intrathecal opioids. *Damevski et al.* reported a significant increase in itching between the intrathecal uses of 0.05 mg and 0.1 mg of morphine [28]. Only the study by *Fenten et al.* demonstrated that, using multimodal analgesia and morphine for PCA, pain scores in THA were low, and the added analgesic value of intrathecal morphine did not outweigh the increased incidence of pruritus [29].

There were several limitations in our study. First, the sample size of the study was relatively small, and this can affect the outcome. To a certain degree, it cannot be ruled out that some side effects of ITM may be underestimated or could have another statistical distribution. Second, the study enrolled healthy patients without respiratory diseases, so the results of this study may not be applicable to patients with respiratory diseases. Third, the patients were followed for only 24 hours after surgery, so this study does not provide data of ITM effects for more than 24 hours. We used a slightly different doses of bupivacaine – from 15 to 18 mg – depending on the patient height, because taller patients need more bupivacaine to achieve the Th10 anaesthesia level.

In the future, more studies are needed to assess the effect of ITM on analgesia and side effects for patients undergoing THA.

## Conclusions

The study results confirmed that adult patients undergoing elective THA under spinal anaesthesia with bupivacaine and 0.2 mg morphine had superior analgesia compared to patients who received spinal analgesia with bupivacaine or bupivacaine and 0.1 mg morphine. The ITM dose of 0.2 mg is safe for patients. While this dose may increase the incidence of pruritus, it does not expose patients to a higher risk of PONV or other systemic side effects.

## Data Availability

The datasets used and analysed in this study may be obtained from the corresponding author upon reasonable request.

## Abbreviations

ASA: American Society of Anesthesiologists
BMI: Body Mass Index
COX: inhibitors of cyclooxygenase
*ERAS*: *Enhanced Recovery After Surgery*
ESRA: European Society of Regional Anaesthesia
IT: intrathecal
ITM: intrathecal morphine
ITM 0.1 mg: Group II
ITM 0.2 mg: Group III
IV: intravenously
NRS: numeric rating scale
NSAIDs: nonsteroidal anti-inflammatory drugs
PACU: post-anaesthesia care unit
PCA: patient-controlled analgesia
P.O.: per os
PONV: postoperative nausea and vomiting
post-op: postoperative
PROSPECT: procedure specific postoperative pain management
R: a language and environment for statistical computing; R Foundation for Statistical Computing, Vienna, Austria)
RR: respiratory rate
SA: spinal anaesthesia
SC: subcutaneously
SpO2: peripheral capillary blood oxygen saturation
THA: total hip arthroplasty
VAS: visual analogue pain scale
